# If You Care About Autonomic Modulation—Do Not Let Seizure Seizure

**DOI:** 10.3390/diagnostics16050698

**Published:** 2026-02-27

**Authors:** Matthias C. Borutta, Vayra Royle, Christina Rothballer, Florian Kraemer, Stephanie Gollwitzer, Hajo Hamer, Stefan Schwab, Julia Koehn

**Affiliations:** 1 Department of Neurology, University of Erlangen-Nuremberg, Schwabachanlage 6, 91054 Erlangen, Germany; vayra.royle@uk-erlangen.de (V.R.); christina.rothballer@uk-erlangen.de (C.R.); florian.kraemer@uk-erlangen.de (F.K.); stefan.schwab@uk-erlangen.de (S.S.); 2 Epilepsy Center, Department of Neurology, University of Erlangen-Nürnberg, Schwabachanlage 6, 91054 Erlangen, Germany; stephanie.gollwitzer@uk-erlangen.de (S.G.); hajo.hamer@uk-erlangen.de (H.H.); 3 Department of Neurology, ANregiomed, Escherichstraße 1, 91522 Ansbach, Germany

**Keywords:** temporal lobe epilepsy, autonomic nervous system, heart rate variability, autonomic dysregulation, dysautonomia

## Abstract

**Background**: To assess associations between possible dysfunction of autonomic cardiovascular modulation and hemispheric localization, seizure frequency, disease duration, and antiseizure medication (ASM) in temporal lobe epilepsy (TLE). **Methods**: In this prospective observational study, cardiovascular autonomic modulation was monitored in 31 patients with TLE (12 patients with right TLE, 19 patients with left TLE). From 5 min time series of R–R intervals (RRI) and blood pressure (BP) recordings, we calculated autonomic parameters of sympathetic, parasympathetic, and total autonomic cardiovascular modulation. Data were compared to those of 30 healthy volunteers. Subgroup analyses were performed according to (1) disease localization (right vs. left hemispheric TLE), (2) seizure frequency (< vs. >1/month) and disease duration (< vs. >10 years), (3) number of ASMs, and (4) participants’ age (< vs. >30 years). **Results**: Between right TLE patients, left TLE patients, and controls, there were no significant differences in the assessed bio-signals. Parameters of sympathetic and total autonomic modulation were slightly lower in right TLE patients than in controls. Additionally, reduced vagal modulation was observed in right TLE patients taking three ASMs or not taking any ASMs at all (applicable to one patient) compared to healthy controls. In general, TLE patients with <1 seizure/month showed lower parameters of sympathetic modulation than healthy controls, with differences reaching statistical significance in left TLE patients. In contrast, parameters reflecting vagal tone showed insignificantly, yet consistently, lower values in left TLE patients with increasing seizure frequency. Alterations in autonomic cardiovascular modulation observed across age-matched subgroups were comparable. **Conclusions**: A trend towards lower values of sympathetic modulation in patients with right TLE supports previous findings suggesting right hemispheric mediation of sympathetic regulation. A decrease in parasympathetic modulation with increasing seizure frequency underscores the importance of sufficient seizure control in order to prevent autonomic complications. In contrast, the absence of significant associations between disease duration and autonomic alterations suggests that epilepsy exerts an early and clinically relevant effect on the autonomic nervous system. Due to comparable alterations in autonomic modulation in a patient without antiseizure medication and in patients undergoing polytherapy, ASM side effects may not account solely for the observed autonomic dysregulation of our TLE patients.

## 1. Introduction

With an approximate incidence of 50–200 per 100,000 inhabitants and a prevalence of 0.5–1.2%, epilepsy represents a significant cause of morbidity and mortality throughout the world [1]. Pathophysiologically, epileptic seizures result from excessive neuronal discharges due to increased neuronal excitability or impaired inhibition [2]. The clinical manifestations of a seizure depend on the brain regions involved. Temporal lobe epilepsy (TLE), also referred to as psychomotor or limbic epilepsy, represents the most common form of focal epilepsy, typically manifesting in childhood or early adulthood [3].

Pharmacological therapy constitutes the mainstay of treatment for TLE. According to current guidelines, treatment should begin with monotherapy [4]. Lamotrigine is recommended as the first-line agent for newly diagnosed focal epilepsy [4], whereas levetiracetam and lacosamide are considered second-line options [4]. In cases where the initial monotherapy fails, a switch of medication or a combination therapy may be considered [4]. Among the various antiepileptic drug classes, sodium channel blockers play a particularly important role—not only because they are commonly used in epilepsy therapy, but also due to their potential cardiac side effects. A shared risk among this class of agents is the occurrence of cardiac arrhythmias, which, especially at higher doses, may manifest as prolongation of the PR interval [5,6].

In addition to the cardiac side effects of antiepileptic treatment, the autonomic nervous system appears to play a crucial role in rhythmogenic events among patients with epilepsy [7]. Autonomic symptoms frequently occur during epileptic seizures due to the extensive anatomical connections between epileptogenic zones and central autonomic structures. Such symptoms often accompany other clinical manifestations and may occur during any phase of a seizure—preictal, ictal, or postictal [7]. Autonomic phenomena are particularly prominent in TLE and in so-called self-limited epilepsy with autonomic seizures (SeLEAS) [7]. Beyond seizure-related autonomic dysfunction, interictal autonomic disturbances are also of major importance in epilepsy. Cardiorespiratory alterations, in particular, are considered key factors contributing to the risk of sudden unexpected death in epilepsy (SUDEP) [8].

Within the literature, several studies have provided evidence for lateralization of the autonomic nervous system, and authors suggested associations between differently impaired autonomic modulation and the affected hemisphere in patients with epilepsy. In 2001, Hilz et al. investigated cardiovascular modulation parameters in a cohort of patients with drug-resistant epilepsy during a Wada test, i.e., before and during hemispheric inactivation. The authors demonstrated that sympathetic activity seems to be predominantly controlled by the right hemisphere, whereas parasympathetic regulation is primarily mediated by the left hemisphere [9]. Other studies reported different findings; yet, although results do not offer a clear picture, most authors consistently conclude that epilepsy-related disruption of autonomic networks may contribute to an elevated risk of cardiac arrhythmias and thereby to an increased risk of SUDEP [10,11,12].

In addition to hemispheric lateralization, other factors—such as antiseizure medication (ASM), disease duration, and seizure frequency—may also impair autonomic cardiovascular modulation in patients with epilepsy. However, the available evidence regarding these associations is largely based on small and heterogeneous patient cohorts, and the reported results are inconsistent [13,14,15,16,17,18,19,20,21].

Therefore, the present study aims to investigate the influence of hemispheric lateralization of TLE on autonomic nervous system function, as well as to explore additional factors that may affect autonomic cardiovascular modulation. Using a well-defined cohort of patients with TLE from a specialized epilepsy center and a matched group of healthy controls, this study seeks to delineate the impact of these variables and to further refine the understanding of potential mechanisms contributing to increased SUDEP risk.

## 2. Materials and Methods

### 2.1. Patient Selection

**Figure 1:**
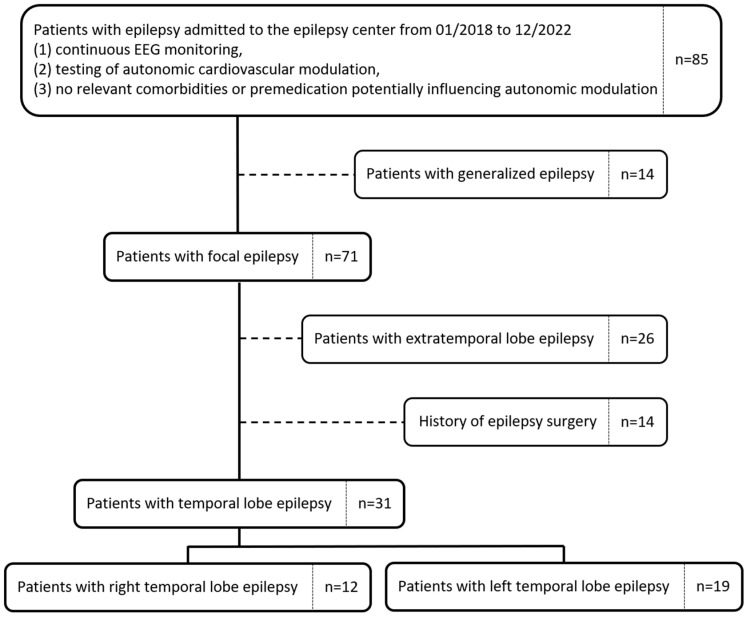
Flowchart of study participants.

All patients admitted to the epilepsy center of the University Hospital Erlangen-Nuremberg, Germany, between January 2018 and December 2022 with previously diagnosed epilepsy were screened for eligibility to participate in the present study. Inclusion criteria consisted of (1) presence of a prolonged video EEG recording, (2) assumed epileptogenic zone located in one temporal lobe based on semiology, EEG, or imaging data, (3) autonomic testing, (4) absence of co-morbidities or medication potentially influencing autonomic modulation, and (5) absence of epilepsy surgery in the past (Figure 1). Testing of autonomic cardiovascular modulation was performed after EEG electrode placement, interictally within 24 h after admission and before reduction of the dosage or any other changes in antiseizure premedication.

In addition, a control group of 30 healthy individuals, comparable in age and sex distribution and without relevant medical history or prior medication, was included in the study.

The study was approved by the ethics committee of the University of Erlangen-Nuremberg, and written informed consent has been obtained from all study participants according to the Declaration of Helsinki.

### 2.2. Clinical and Epilepsy Parameters

Diagnosis of TLE was made by the treating physicians, as described elsewhere [22]. After hospital admission, individuals underwent presurgical long-term scalp EEG recordings. EEG was recorded digitally at a sampling rate of 256 Hz. For review, filter settings were kept at 0.5 to 70 Hz bandpass and sensitivity at 150 μV/cm. We retrieved data on demographic parameters (age and sex), prior comorbidities (hypertension, diabetes mellitus, congestive heart failure, etc.), relevant premedication, as well as current medication, disease duration, and seizure frequency from the institutional electronic databases. Disease duration was classified as either >10 years or <10 years, and seizure frequency was categorized as daily, weekly, monthly, yearly, or rare/unknown number of seizures. Patients were then divided into two subgroups according to the affected hemisphere, i.e., right-hemispheric TLE or left-hemispheric TLE (Table 1).

### 2.3. Parameters of Autonomic Cardiovascular Modulation

When measuring autonomic cardiovascular modulation, a distinction can be made between short-term and long-term measurements depending on the hypothesis being investigated. While long-term recordings often comprise a minimum duration of 18 to 24 h, short-term recordings cover a period of a few minutes (usually at least two to five minutes) [23]. Long-term recordings, due to their measurement duration, provide a better picture of the numerous environmental influences (e.g., changes in outside temperature) and the body’s own processes (e.g., minor fluctuations in autonomic modulation due to circadian rhythms) on the modulation of the cardiovascular system [23]. Short-term recordings, on the other hand, are characterized by a low number of measurement artifacts [23]. In order to obtain a picture of the current autonomous cardiovascular modulation and, if applicable, autonomic dysfunction, a short recording of 5 min has therefore been chosen for the purpose of our study. To ensure comparable measurement conditions and to account for potential circadian variations in autonomic modulation, all measurements were conducted in a quiet room at an average ambient temperature of 24 °C and stable humidity, between 9:00 a.m. and 2:00 p.m. After a 40 min resting period in a seated position to ensure stable cardiovascular conditions, the test setup and the assessment of autonomic modulation were performed. Parameters of cardiovascular autonomic modulation were monitored using 3-lead electrocardiography (with a sampling rate of 200 Hz) for a 5 min time series of R–R intervals (RRI) and non-invasive beat-to-beat blood pressure measurement via finger pulse photoplethysmography (Portapres ^®^, TPD Biomedical Instrumentation, Amsterdam, The Netherlands) on the index or middle finger (calibrated against the ipsilateral brachial artery BP) [24]. Both biosignals were recorded simultaneously and continuously for a period of 5 min. Data acquisition, artifact correction, further processing, and analysis of the digitized data were carried out using a custom-designed data acquisition and analysis system (SUEmpathy™, SUESS Medizin-Technik GmbH, Aue, Germany) [23].

In the next step, the parameters were analyzed in both the time and frequency domains.

Parameters in the so-called time domain were calculated from the tachogram of the RRIs and reflect different aspects of autonomic modulation: the standard deviation of RRIs (SD) as an absolute measure of dispersion and the coefficient of variation (CV) as a relative measure of dispersion reflect both sympathetic and parasympathetic influences on autonomic cardiovascular modulation, thus representing an overall measure of autonomic control. Furthermore, the root mean square of successive differences between adjacent RRIs (RMSSD), calculated in milliseconds, reflects exclusively parasympathetic influences on heart rate variability [23,24]. There are no established linear analysis methods to determine specific sympathetic parameters in the time domain.

Complementary analyses in the frequency domain allow for a more detailed investigation of spontaneous oscillations in autonomic modulation, thereby revealing subtle changes in autonomic cardiovascular function [23,24]. Various mathematical models are available for spectral analysis; in this study, trigonometric regressive spectral analysis (TRS) was used [23]. The TRS algorithm utilizes short segments of digitized biosignals with a duration of 20–30 s, which are continuously analyzed in successive time series. By recording short segments of 2–5 min, spectral analysis allows for the decomposition of slow underlying frequencies into three main components: very low frequency (VLF, <0.04 Hz), low frequency (LF, 0.04–0.15 Hz), and high frequency (HF, 0.15–0.4 Hz). The spectral power within each frequency range is calculated as the area under the curve of the power spectral density function and expressed as an integral in ms^2^/Hz [23,24].

Fluctuations within the HF range of the RRI (RRI-HF power) reflect almost exclusively parasympathetic activity mediated by the vagus nerve, whereas the LF range (RRI-LF power) predominantly represents sympathetic modulation, although vagal influences, particularly at rest, may also contribute [23,24]. The sum of spectral powers across all frequency bands, the so-called RRI total power, represents a measure of overall autonomic cardiovascular modulation [23,24]. The ratio of LF to HF power (LF/HF ratio) is used as an indicator of the balance between sympathetic and parasympathetic modulation [23,24].

According to the literature, spectral analysis of the LF component of blood pressure is considered a reliable indicator of sympathetic tone [23,24]. In contrast, the HF component of systolic blood pressure reflects not only parasympathetic influences but also respiration-dependent fluctuations in venous return and changes in cardiac output; therefore, this parameter provides only limited information on autonomic modulation and was not calculated in the present study [23,24].

### 2.4. Statistical Analysis

For data analysis, a commercially available statistical program (IBM SPSS Statistics for Windows, version 24) was used, and significance was set at *p*  <  0.05. Testing for normal distribution was performed using the Kolmogorov–Smirnov test. Data are expressed as mean and standard deviation or median and interquartile range, according to distribution.

Normally distributed patient and control data were compared using the *t*-test for unpaired samples and the Mann–Whitney U test for comparison of non-normally distributed data.

Nominally scaled variables were compared using the chi-square test of associations and, in cases with small sample sizes, using the Fisher exact probability test.

For further analyses, i.e., hemispheric lateralization, impact of seizure frequency and disease duration, ASM, or age on autonomic modulation, patients were categorized according to:

1. Disease localization, i.e., right- or left hemispheric TLE
2. Seizure frequency < 1 vs. >1/month, and disease duration < 10 vs. >10 years
3. Number of ASM
4. Age, i.e., individuals younger than 30 years and those older than 30 years

## 3. Results

### 3.1. Baseline Characteristics

Over a 5-year period, 84 patients with presumed epilepsy were screened for eligibility (Figure 1). After exclusion of patients who failed diagnostic criteria for focal epilepsy, lacked assessment of autonomic cardiovascular modulation, had co-morbidities, or were premedication known to potentially influence autonomic modulation, 71 patients with focal epilepsy were enrolled in the study (29 women, 42 men). A diagnosis of TLE, i.e., the epileptogenic zone located in the temporal lobe based on semiology, EEG, or imaging data was made in 31 patients (mean age 36 years [27–46 years]; 30.8% female). Twelve patients were diagnosed with right hemispheric TLE (5 women; 37.5 (28.0–56.5) years) and 19 patients were diagnosed with left hemispheric TLE (11 women; 32.0 (25.0–43.0) years). Parameters of cardiovascular autonomic modulation of patients were compared to data from 30 healthy volunteers (16 women; 38.0 (28.0–52.0) years).

Clinical baseline characteristics of both patient subgroups, i.e., right and left hemispheric TLE, and controls are presented in Table 1. There was no significant difference between the two patient groups regarding age, sex, disease duration, seizure frequency, and antiseizure medication.

### 3.2. Association Between Autonomic Modulation and Hemispheric Localization of TLE

Overall, between right TLE patients, left TLE patients, and controls, there were no significant differences in the assessed bio-signals used for further analysis of autonomic modulation, i.e., RRIs, mean SBP, and mean DBP (Table 2).

Parameters of vagal cardiac modulation (RRI-RMSSD, RRI-HF powers) did not differ between both patient groups and controls (Table 2).

**Figure 2:**
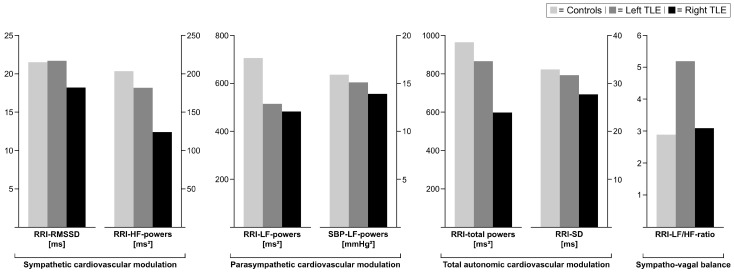
Bar charts of median values of autonomic cardiovascular modulation according to hemispheric localization. *TLE*, temporal lobe epilepsy; *RRI*, RR interval; *RMSSD*, root mean square of successive differences; *HF*, high frequency; *LF*, low frequency; *SBP*, systolic blood pressure; *SD*, standard deviation.

In contrast, parameters reflecting sympathetic cardiac modulation, i.e., RRI-LF powers, were slightly lower in right TLE patients than in controls (Table 2, Figure 2).

Similarly, right TLE patients had slightly, yet not significantly, lower RRI-SDs (i.e., values of parameters of total autonomic modulation) and RRI-LF/HF ratios (i.e., index of sympatho-vagal balance) than controls (Table 2, Figure 2).

All assessed parameters of autonomic cardiovascular modulation did not differ between patients with right and left TLE (Table 2).

### 3.3. Association Between Autonomic Modulation, Disease Duration, and Seizure Frequency

Disease duration and seizure frequency of all patients are summarized in Table 1. Regarding disease duration, right and left TLE patients were classified as <10 years vs. >10 years of established diagnosis. Seven patients had an established diagnosis of right TLE < 10 years (58.3%), five patients had right TLE > 10 years (41.7%), six patients had left TLE < 10 years (31.6%), and thirteen patients had left TLE > 10 years (68.4%).

Parameters of vagal cardiac modulation (RRI-RMSSD, RRI-HF powers) again did not differ between patient groups and controls (Table 3).

RRI-LF powers, RRI-SDs, and RRI total powers were slightly lower in right TLE patients with disease duration < 10 years than in controls, whereas these values did not differ between right TLE patients with disease duration > 10 years, left TLE patients with disease duration < 10 years, left TLE patients with disease duration > 10 years, and controls (Table 3).

Sympatho-vagal balance, i.e., RRI-LF/HF ratios, was slightly lower in right TLE patients with disease duration > 10 years than in controls (Table 3).

Regarding seizure frequency, right and left TLE patients were classified as < 1/month vs. >1/month.

Six right TLE patients had <1/month (50.0%), six right TLE patients had >1/month (50.0%), 10 left TLE patients had <1/month (52.6%), and nine left TLE patients had >1/month (47.4%).

Statistically significant associations between autonomic modulation and seizure frequency were only seen in SBP-LF powers: SBP-LF powers were significantly lower in left TLE patients with <1 seizure per month than in healthy controls, and slightly, yet not significantly lower in right TLE patients with <1 seizure per month than in healthy controls (Table 3). In contrast, parameters reflecting vagal tone showed insignificantly, yet consistently lower values in left TLE patients with increasing seizure frequency, whereas sympathovagal balance increased slightly in this patient group.

### 3.4. Association Between Autonomic Modulation and Antiseizure Medication

A detailed overview of ASM in right and left TLE patients is summarized in Table 1. In our cohort, most patients had a combination of two ASMs at the time of assessment (5 right TLE patients, 41.7%; 11 left TLE patients, 57.9%). In both groups, 4 patients were on 3 different ASMs; only 2 right TLE (16.7%) and 4 left TLE patients (21.1%) were on a mono-therapy, and 1 patient with right TLE (8.3%) did not take any ASM.

Due to small cohort sizes, statistical results of subgroup analyses regarding the association between autonomic modulation and ASM are rather limited. Yet, we found lower values of parameters reflecting sympathetic cardiac modulation (RRI-LF powers, SBP-LF powers), RRI-RMSSDs (i.e., a parameter of vagal cardiac modulation), values of parameters of total autonomic modulation (RRI-SDs, RRI-CVs, RRI total powers), and RRI-LF/HF ratios in right TLE patients who took 3 different ASMs at the time of assessment, as well as in our one right TLE patient without any ASM, than in healthy controls Table 4.

### 3.5. Association Between Autonomic Modulation and Age

In a final analysis, both patient subgroups and the healthy control group were further divided into subgroups based on age: individuals younger than 30 years and those older than 30 years.

In the control group, subdivision by age revealed significantly lower values in the older cohort compared to the younger participants for sympathetic modulation (RRI-LF power), parasympathetic modulation (RRI-HF power), and total autonomic modulation (RRI-SD, RRI-CV, and RRI total power) (Table 5). Similar trends were observed when comparing younger and older right TLE patients, as well as younger and older left TLE patients.

Patients with left-hemispheric TLE younger than 30 years exhibited significantly lower values of sympathetic modulation (RRI-LF power) and total autonomic modulation (RRI-CV and RRI total power) compared to age-matched healthy controls (Table 5).

In patients with right-hemispheric TLE, a trend toward lower values was observed in the subgroup older than 30 years compared with age-matched controls. This trend was evident for parameters of total autonomic modulation (RRI-SD, RRI-CV, RRI total power) as well as for sympathetic modulation (RRI-LF power) (Table 5).

Additional analyses performed for age cutoffs of >/<40 years and >/<50 years did not reveal any further relevant findings.

## 4. Discussion

### 4.1. Summary of Key Findings

The present study yielded the following relevant findings:

1. A trend toward lower values of sympathetic modulation in patients with right TLE suggests that the localization of the epileptic focus influences autonomic cardiovascular modulation, with structural alterations in sympathetic regulation predominantly mediated by the right hemisphere.
2. Given the comparable alterations in autonomic cardiovascular modulation observed across age-matched subgroups, the differences between epilepsy patients and healthy controls cannot be explained solely by physiological, age-associated changes.
3. A decrease in parasympathetic modulation with increasing seizure frequency underscores the importance of sufficient seizure control in order to prevent autonomic complications. The absence of significant associations between disease duration and alterations in autonomic modulation suggests that epilepsy exerts an early and clinically relevant effect on the autonomic nervous system.
4. Although the interpretability is limited by the small sample size, the findings indicate that antiseizure medication alone does not account for the observed autonomic dysregulation in epilepsy patients. Comparable alterations in autonomic modulation were observed both in a patient without antiepileptic medication and in patients undergoing polytherapy with at least three different agents.

### 4.2. Influence of TLE Localization on Autonomic Cardiovascular Modulation

Lateralization is a fundamental principle in cerebral organization. It is well known that motor function is controlled contralaterally and that, in the majority of individuals, language function is localized within the left hemisphere. Similar to other neural systems, lateralization of the autonomic nervous system has also been postulated in several studies.

In an anesthesiological study by Hilz et al., intra-arterial administration of amobarbital into the right internal carotid artery induced functional inactivation of the right hemisphere, which resulted in an increase in parasympathetic activity [9]. Based on these findings, the authors concluded that sympathetic activity is predominantly governed by the right hemisphere. However, due to the global inactivation of the hemisphere, precise structural localization was not possible in that study [9].

Subsequent investigations examining patients with TLE have yielded partially conflicting results. Several studies reported increased sympathetic activity or reduced parasympathetic activity in patients with rTLE [10,12].

In the present study, patients with rTLE showed a trend toward lower values of sympathetic modulation in the interictal state. Assuming that epilepsy induces structural and connective alterations, these data align with the findings of Hilz et al. Conversely, some previous studies, e.g., by Dono et al., observed increased sympathetic activity in rTLE during interictal EEG abnormalities [12]. This may reflect transient hemispheric hyperactivity during interictal epileptiform discharges. Under this assumption, the data of Dono et al. would not contradict the current findings but rather support the concept that sympathetic modulation is primarily controlled by the right hemisphere.

Beyond epilepsy, lateralization of central autonomic control has also been demonstrated in other neurological conditions. Studies of ischemic stroke patients revealed a higher risk of cardiac arrhythmias following right-hemispheric strokes, particularly when the insula or amygdala are involved [25,26]. These findings suggest that either heightened sympathetic tone or reduced parasympathetic activity contributes to arrhythmogenesis.

Experimental data further support these clinical observations. In an animal model, injection of autologous blood into the right insular region of rats induced sympathetic cardiac alterations, presumably via activation of downstream structures such as the dorsomedial hypothalamus [26].

Taken together, both previous studies and the present findings suggest a lateralization of the central autonomic nervous system, with the temporal lobe and insular cortex playing key roles in this network. These effects have been observed across different disease entities, including epilepsy and stroke. However, the precise pathophysiological mechanisms underlying sympathetic and parasympathetic alterations following structural or functional damage within the central autonomic network remain unclear. Further studies are needed to clarify these mechanisms, particularly with respect to increased risks of SUDEP, and to identify potential therapeutic implications.

### 4.3. Influence of (Patient) Age on Autonomic Cardiovascular Modulation

With increasing age, autonomic cardiovascular modulation shifts toward sympathetic predominance, whereas younger and physically active individuals exhibit higher baseline vagal tone [27]. Age is also one of the strongest independent risk factors for the development of cardiovascular disease.

In this study, the observed changes in autonomic cardiovascular modulation in both the patient cohort and the control group aged over 30 years reflect these physiological age-related changes. These findings were consistent across all subgroups, without clear differences between the respective patient and control cohorts. Therefore, the additional alterations in autonomic modulation observed in this study cannot be attributed solely to physiological, age-associated variation.

### 4.4. Influence of Seizure Frequency and Disease Duration on Autonomic Cardiovascular Modulation

Epileptic seizures are known to cause autonomic alterations across multiple systems, including cardiovascular, respiratory, gastrointestinal, urogenital, and cutaneous domains. Previous studies have also demonstrated an increased risk of interictal autonomic dysregulation in patients with TLE [10,12,13].

Ansakorpi et al. compared patients with well-controlled epilepsy and those with drug-resistant epilepsy, showing signs of autonomic dysregulation in the latter group [13]. The authors concluded that disease severity and seizure frequency correlate with changes in autonomic cardiovascular modulation [13]. Although studies examining ictal autonomic changes have suggested variable results, most authors agree that long-standing epilepsy is associated with predominant parasympathetic dysfunction in the interictal period.

In the present study, patients with left-hemispheric TLE exhibited a decrease in vagal tone, but no significant differences were found between subgroups with short and long disease duration. Furthermore, among patients with left TLE, higher seizure frequency was associated with an increase in sympathovagal balance, indicating a relative reduction in parasympathetic activity. These findings are consistent with previous reports demonstrating diminished parasympathetic modulation in drug-resistant epilepsy [28].

In contrast, no significant correlations were observed between disease duration and autonomic parameters, suggesting that the impact of epilepsy on autonomic regulation may occur early in the disease course. This interpretation is supported by findings from Goit et al., who identified increased sympathetic and reduced parasympathetic activity even in newly diagnosed epilepsy patients [14]. Similarly, Ansakorpi et al. concluded that epileptic activity, rather than disease duration, exerts a stronger influence on autonomic modulation [15].

The underlying pathophysiology remains unclear. Dütsch et al. found no evidence that structural lesions alone explain the observed changes [16]. Subclinical alterations such as interictal epileptiform activity, which may precede the first clinically manifest seizures and thus the diagnosis of epilepsy, may already contribute to early autonomic dysregulation.

### 4.5. Influence of Antiseizure Medication on Autonomic Cardiovascular Modulation

ASMs, particularly sodium channel blockers, are known to have adverse cardiac effects. Carbamazepine and phenytoin are frequently cited in this context [18,19,21]. Reported side effects include bradyarrhythmias such as atrioventricular block and asystole, especially in cases of overdose [13]. For phenytoin, relevant cardiac effects occur mainly after intravenous administration. Sudden discontinuation of carbamazepine has also been associated with increased nocturnal sympathetic activity [17].

Studies examining the influence of ASMs on autonomic modulation have suggested inconsistent results. Some investigations found reduced HRV in patients taking carbamazepine compared to untreated controls, whereas others observed no significant change during long-term monotherapy [13,18,19].

While most previous studies examined patients already receiving ASMs, Persson et al. analyzed HRV before and after initiation of carbamazepine therapy and demonstrated a decrease in HRV following treatment onset, suggesting a medication-related effect [19].

Data on newer ASMs are limited. A study investigating patients treated with perampanel reported increased HRV compared to controls, suggesting a possible cardioprotective effect [20]. In contrast, no significant HRV changes were observed in patients receiving lacosamide monotherapy [21].

In the present study, the overall influence of antiepileptic medication on autonomic modulation was analyzed, followed by subgroup analyses focusing on sodium channel blockers. Compared with the control group, patients with rTLE taking three ASMs, as well as one unmedicated patient with rTLE, showed lower values for sympathetic modulation (RRI-LF power, SBP-LF power), parasympathetic modulation (RRI-RMSSD), and total autonomic modulation (RRI-SD, RRI-CV, RRI total power, RRI LF/HF ratio). Similar alterations were observed in patients with rTLE treated with sodium channel blockers.

These findings support the notion that antiseizure medication alone does not fully explain autonomic dysregulation in epilepsy. Compared with healthy controls, both medicated and unmedicated patients exhibited lower values across multiple autonomic parameters. However, because only one patient in our cohort was ASM naive, any possible correlations between the absence of drug therapy and autonomic dysfunction remain highly speculative. Consequently, antiseizure medication might exert an indirect protective effect by reducing seizure frequency. Nevertheless, potential medication-induced alterations in autonomic modulation, particularly under polytherapy, should be carefully considered. Further studies assessing autonomic function prior to and during treatment could help identify individual medication effects and guide therapeutic adjustments based on risk profiles.

### 4.6. SUDEP

The pathophysiology behind SUDEP remains incompletely understood. It is thought to involve a combination of cardiac arrhythmia, respiratory failure, and brainstem dysfunction occurring in the postictal phase [29,30,31]. In particular, some patients with refractory epilepsy have been found to have elevated IgG levels and antibodies that target different glutamate receptor peptides, leading to neuronal loss and persistent epileptic seizures. Therefore, immune-mediated mechanisms may be considered to play a substantial role in SUDEP [32]. The most prominent risk factors include high seizure frequency and drug-resistant epilepsy.

An autonomic component in the mechanism of SUDEP has been widely discussed, underscoring the relevance of identifying factors contributing to autonomic dysregulation in epilepsy patients. Since SUDEP often follows unwitnessed nocturnal seizures, data remain limited.

In the MORTEMUS study, 11 cases of SUDEP were analyzed among patients undergoing in-hospital EEG monitoring [33]. In nine of these cases, concurrent ECG data were available. The sequence of events was similar across cases: postictal loss of consciousness with respiratory insufficiency and transient, self-limiting tachyarrhythmia, followed by generalized EEG suppression, bradycardia, and subsequent asystole [33]. These observations suggest that postictal sympathetic activation occurs initially, while the fatal phase likely results from a combination of elevated vagal tone and central hypoventilation [33].

Although MORTEMUS patients mainly represented individuals with therapy-resistant epilepsy, and therefore not the general epilepsy population, similar results have been replicated in animal models [26]. In summary, the mechanisms underlying SUDEP appear multifactorial. Yet, regardless of the precise mechanism, seizure frequency reduction remains the key preventive strategy. This conclusion is also supported by the present findings, which indicate that adequate seizure control is associated with less pronounced alterations in autonomic modulation. Clinically, this underscores the importance of considering further therapeutic options, such as epilepsy surgery, in cases of insufficient pharmacological seizure control.

### 4.7. Limitations

Despite our findings, several limitations must be mentioned. Overall, the study includes a rather small cohort, which limits the statistical power of the subgroup analyses in particular. This aspect is evident when analyzing and interpreting the influence of ASM on autonomic dysfunction. Although sodium channel blockers were used most frequently, the high percentage of heterogeneous polytherapy as well as the number of different substance class medications limits the interpretable significance of the influence of ASM on autonomic modulation. Furthermore, the limited number of patients does not allow for a more detailed breakdown of seizure frequency and disease duration within the analyses. Despite various preliminary studies, only statistical trends and no significant differences between right-sided and left-sided TLE could be described. A larger cohort and longitudinal data analysis are still needed to better validate the selected cutoffs for seizure frequency and disease duration and to add further cutoffs, such as daily, weekly, and monthly seizure frequency. Finally, further clinical parameters such as validated risk markers or a longitudinal follow-up are lacking for a clear statement on dysautonomia in SUDEP.

Until further studies with larger cohorts and possibly also ictal and postictal measurements are conducted, our exploratory data nevertheless reveal important findings in connection with various clinically relevant parameters associated with autonomic dysfunction in TLE.

### 4.8. Conclusions

Besides all mentioned limitations, our results indicate that patients with TLE exhibit alterations in autonomic cardiovascular modulation. Our results underscore the importance of sufficient seizure control in order to prevent autonomic complications and fatalities.

## Figures and Tables

**Table 1:** Baseline characteristics of healthy controls and 31 patients with temporal lobe epilepsy according to hemispheric localization.

	Controls (n = 30)	All Patients with TLE (n = 31)	Patients with rTLE (n = 12)	Patients with lTLE (n = 19)	*p*-Values (rTLE vs. lTLE)
**Age** [years], Median (IQR)	38.0 (28.0–52.0)	36.0 (27.0–46.0)	37.5 (28.0–56.5)	32.0 (25.0–43.0)	0.22
**Age split**	-				
<30 years, n (%)		11 (35.5)	3 (25.0)	8 (42.1)	0.45
<30 years, Median (IQR)		25.0 (21.0–28.0)	27.0 (-)	23.5 (21.0–28.8)	0.61
≥30 years, n (%)		20 (64.5)	9 (75.0)	11 (57.9)	0.45
≥30 years, Median (IQR)		42.0 (36.3–52.0)	51.0 (35.5–61.5)	41.0 (37.0–46.0)	0.52
**Female sex**, n (%)	16 (53.3)	16 (51.6)	5 (41.7)	11 (57.9)	0.47
**Disease duration**	-				
<10 years, n (%)		13 (41.9)	7 (58.3)	6 (31.6)	0.26
≥10 years, n (%)		18 (58.1)	5 (41.7)	13 (68.4)	0.26
**Seizure frequency**	-				
daily, n (%)		2 (6.5)	1 (8.3)	1 (5.3)	1
weekly, n (%)		3 (9.7)	1 (8.3)	2 (10.5)	1
monthly, n (%)		10 (32.3)	4 (33.4)	6 (31.6)	1
yearly, n (%)		7 (22.6)	3 (25.0)	4 (21.1)	1
rarely/unknown, n (%)		8 (25.8)	3 (25.0)	6 (31.6)	1
**Number of ASM**	-				
none, n (%)		1 (3.2)	1 (8.3)	0 (0.0)	0.39
1 ASM, n (%)		6 (19.4)	2 (16.7)	4 (21.1)	1
2 ASM, n (%)		16 (51.6)	5 (41.7)	11 (58.0)	0.47
3 ASM, n (%)		8 (25.8)	4 (33.3)	4 (21.1)	0.68
**ASM premedication**	-				
SCB, n (%)		23 (74.2)	9 (75.0)	14 (73.7)	1
Dual SCB therapy, n (%)		8 (25.8)	3 (25.0)	5 (26.3)	1
Levetiracetam, n (%)		15 (58.4)	5 (25.0)	10 (52.6)	0.72
Brivaracetam, n (%)		2 (6.5)	1 (8.3)	1 (5.3)	1
Valproate, n (%)		2 (6.5)	0 (0.0)	2 (10.5)	0.51
Topiramate, n (%)		1 (3.2)	0 (0.0)	1 (5.3)	1
Zonisamide, n (%)		4 (12.9)	3 (25.0)	4 (21.1)	1
Perampanel, n (%)		1 (3.2)	0 (0.0)	1 (5.3)	1
Pregabalin, n (%)		1 (3.2)	1 (8.3)	0 (0.0)	0.39
**SCBs**	-				
Lamotrigine, n (%)		12 (38.7)	4 (33.4)	8 (42.1)	0.72
Lacosamide, n (%)		12 (38.7)	5 (41.7)	7 (36.8)	1
Carbamazepine, n (%)		1 (3.2)	1 (8.3)	0 (0.0)	0.39
Oxcarbazepine, n (%)		5 (16.1)	1 (8.3)	4 (21.1)	0.63
Eslicarbazepine, n (%)		4 (12.9)	3 (25.0)	1 (5.3)	0.27
**Drug-resistant epilepsy**, n (%)		21 (67.7)	9 (75.0)	12 (63.2)	0.70

*TLE*, temporal lobe epilepsy; *rTLE*, right TLE; *lTLE*, left TLE; *ASM*, antiseizure medication; *SCB*, sodium channel blocker.

**Table 2:** Biosignals and parameters of autonomic cardiovascular modulation according to hemispheric localization.

	Controls (n = 30)	Patients with rTLE (n = 12)	Patients with rTLE (n = 19)	*p*-Values (Ct vs. lTLE)	*p*-Values (Ct vs. lTLE)	*p*-Values (rTLE vs. lTLE)
**Biosignals**, Median (IQR)						
mean RRI [ms]	754.1 (723.9–865.9)	727.9 (647.2–847.2)	787.4 (675.5–847.1)	1.0	0.22	0.50
mean SBP [ms]	127.2 (122.0–139.3)	120.8 (113.0–139.4)	121.7 (112.6–130.7)	0.05	0.33	0.63
**Parasympathetic modulation**						
RRI-RMSSD [ms]	21.5 (17.2–35.6)	18.2 (7.5–29.6)	21.7 (11.4–29.3)	0.31	0.15	0.72
RRI-HF powers [ms^2^]	203.4 (85.3–498.1)	124.0 (29.0–450.5)	181.7 (38.5–407.0)	0.47	0.32	0.87
**Sympathetic modulation**						
RRI-LF powers [ms^2^]	705.7 (362.2–2024.3)	482.4 (98.4–837.0)	514.9 (316.1–1409.4)	0.23	0.07	0.47
SBP-LF powers [mmHg^2^]	15.9 (9.9–30.8)	13.9 (6.2–26.2)	15.1 (6.6–21.4)	0.23	0.30	0.81
**Total autonomic modulation**						
RRI total powers [ms^2^]	964.6 (437.3–2521.8)	597.8 (145.8–1352.0)	866.0 (360.5–1506.8)	0.33	0.10	0.54
RRI-SD [ms]	32.9 (24.0–52.9)	27.7 (13.3–36.6)	31.7 (21.4–43.0)	0.34	0.09	0.52
RRI-CV [%]	4.4 (3.2–6.7)	3.3 (2.2–4.8)	4.1 (2.7–5.1)	0.24	0.11	0.52
**Sympatho-vagal balance**						
RRI-LF/HF-ratio	2.9 (1.8–4.5)	3.1 (1.5–7.5)	5.2 (2.4–10.4)	0.25	0.08	0.72

*rTLE*, right TLE; *lTLE*, left TLE; *Ct*, controls; *RRI*, RR interval; *SBP*, systolic blood pressure; *RMSSD*, root mean square of successive differences; *HF*, high frequency; *LF*, low frequency; *SD*, standard deviation; *CV*, coefficient of variation.

**Table 3:** (**a**) Biosignals and parameters of autonomic cardiovascular modulation according to disease duration. (**b**) Biosignals and parameters of autonomic cardiovascular modulation according to seizure frequency.

(**a**)																		
		**Controls** (n = 30)			**Patients with rTLE <10 a** (n = 7)				**Patients with rTLE > 10 a** (n = 5)			**Patients with lTLE < 10 a** (n = 6)				**Patients with lTLE > 10 a** (n = 13)		
**Biosignals**, Median (IQR)																		
mean RRI [ms]		754.1 (723.9–865.9)			730.6 (573.7–856.3)				725.2 (678.7–903.7)			757.9 (669.4–842.3)				793.4 (663.4–921.9)		
mean SBP [ms]		127.2 (122.0–139.3)			125.4 (104.4–144.8)				115.8 (113.4–136.2)			126.5 (119.4–130.2)				118.8 (107.1–131.1)		
**Parasympathetic modulation**																		
RRI-RMSSD [ms]		21.5 (17.2–35.6)			15.6 (7.4–22.2)				23.9 (6.8–33.5)			17.1 (6.7–24.5)				22.6 (11.6–30.8)		
RRI-HF powers [ms^2^]		203.4 (85.3–498.1)			63.1 (45.8–239.1)				301.5 (16.9–665.6)			94.3 (17.6–435.5)				189.0 (41.5–390.4)		
**Sympathetic modulation**																		
RRI-LF powers [ms^2^]		705.7 (362.2–2024.3)			371.7 (129.0–872.1)				673.6 (79.9–1635.6)			644.3 (58.8–1582.8)				514.9 (357.7–1356.1)		
SBP-LF powers [mmHg^2^]		15.9 (9.9–30.8)			12.8 (6.3–26.1)				19.9 (4.7–42.2)			9.6 (5.5–22.6)				17.5 (6.8–21.1)		
**Total autonomic modulation**																		
RRI total powers [ms^2^]		964.6 (437.3–2521.8)			556.7 (152.4–1111.2)				1033.1 (96.8–2272.1)			901.8 (76.4–1773.4)				790.1 (454.9–1611.2)		
RRI-SD [ms]		32.9 (24.0–52.9)			26.2 (14.5–35.2)				33.4 (11.6–49.2)			33.0 (10.0–46.7)				31.5 (23.7–41.3)		
RRI-CV [%]		4.4 (3.2–6.7)			3.2 (2.2–4.8)				3.4 (1.7–6.7)			4.5 (1.4–5.8)				4.0 (2.8–5.5)		
**Sympatho-vagal balance**																		
RRI-LF/HF-ratio		2.9 (1.8–4.5)			3.8 (1.7–4.7)				2.3 (1.7–3.9)			2.3 (1.3–5.0)				5.0 (1.6–10.6)		
		***p*****-Values** (Ct vs. rTLE < 10 a)		***p*****-Values** (Ct vs. rTLE > 10 a)			***p*****-Values** (Ct vs. lTLE < 10 a)				***p*****-Values** (Ct vs. lTLE > 10 a)			***p*****-Values** (rTLE < 10 a vs. >10 a)			***p*****-Values** (lTLE < 10 a vs. >10 a)	
**Biosignals**, Median (IQR)																		
mean RRI [ms]		0.28		0.45			0.64				0.71			0.69			0.54	
mean SBP [ms]		0.64		0.28			0.45				0.04			0.69			0.34	
**Parasympathetic modulation**																		
RRI-RMSSD [ms]		0.11		0.60			0.13				0.71			0.89			0.88	
RRI-HF powers [ms^2^]		0.19		0.93			0.22				0.87			0.89			0.96	
**Sympathetic modulation**																		
RRI-LF powers [ms^2^]		0.07		0.35			0.37				0.33			0.89			0.73	
SBP-LF powers [mmHg^2^]		0.26		0.78			0.14				0.54			0.78			0.66	
**Total autonomic modulation**																		
RRI total powers [ms^2^]		0.09		0.45			0.37				0.46			0.78			0.81	
RRI-SD [ms]		0.09		0.42			0.14				0.44			0.76			0.73	
RRI-CV [%]		0.19		0.28			0.42				0.32			0.76			0.66	
**Sympatho-vagal balance**																		
RRI-LF/HF-ratio		0.33		0.07			0.14				0.58			0.25			0.4	
(**b**)																		
		**Controls** (n = 30)			**Patients with rTLE < 1/M** (n = 6)				**Patients with rTLE > 1/M** (n = 6)			**Patients with lTLE < 1/M** (n = 9)				**Patients with lTLE > 1/M** (n = 10)		
**Biosignals**, Median (IQR)																		
mean RRI [ms]		754.1 (723.9–865.9)			771.2 (566.8–891.1)				717.2 (648.0–841.6)			743.8 (607.9–841.3)				801.2 (759.6–907.6)		
mean SBP [ms]		127.2 (122.0–139.3)			120.8 (113.0–131.6)				127.9 (110.5–146.1)			120.0 (107.1–130.1)				124.9 (115.9–131.8)		
**Parasympathetic modulation**																		
RRI-RMSSD [ms]		21.5 (17.2–35.6)			14.3 (7.0–36.0)				18.9 (9.8–25.8)			23.3 (14.6–32.1)				18.6 (7.1–25.6)		
RRI-HF powers [ms^2^]		203.4 (85.3–498.1)			122.7 (17.5–518.3)				151.1 (40.2–415.8)			224.1 (79.5–382.1)				97.4 (28.7–444.3)		
**Sympathetic modulation**																		
RRI-LF powers [ms^2^]		705.7 (362.2–2024.3)			2230.0 (80.3–1878.2)				633.3 (215.5–766.7)			530.2 (378.5–1030.3)				514.9 (162.0–1704.2)		
SBP-LF powers [mmHg^2^]		15.9 (9.9–30.8)			8.4 (5.0–33.5)				17.5 (11.2–26.8)			7.4 (5.7–15.3)				19.8 (16.3–23.0)		
**Total autonomic modulation**																		
RRI total powers [ms^2^]		964.6 (437.3–2521.8)			350.1 (101.6–2396.5)				836.0 (268.7–1191.5)			828.0 (531.4–1324.5)				937.7 (192.7–1985.2)		
RRI-SD [ms]		32.9 (24.0–52.9)			21.8 (11.8–58.4)				29.8 (17.6–35.7)			32.9 (25.1–38.0)				31.7 (15.7–48.6)		
RRI-CV [%]		4.4 (3.2–6.7)			2.7 (1.7–7.5)				4.0 (2.3–4.7)			4.1 (2.7–5.2)				4.3 (2.8–5.8)		
**Sympatho-vagal balance**																		
RRI-LF/HF-ratio		2.9 (1.8–4.5)			3.5 (1.5–6.2)				2.9 (1.8–3.9)			2.9 (1.2–5.2)				5.3 (1.6–14.5)		
	***p*****-Values** (Ct vs. rTLE < 1/M)		***p*****-Values** (Ct vs. rTLE > 1/M)			***p*****-Values** (rTLE < 1 vs. >1/M)		***p*****-Values** (Ct vs. lTLE < 1/M)		***p*****-Values** (Ct vs. lTLE > 1/M)			***p*****-Values** (lTLE < 1 vs. >1/M)		***p*****-Values** (<1/M r vs. lTLE)			***p*****-Values** (>1/M r vs. lTLE)
**Biosignals**, Median (IQR)																		
mean RRI [ms]	0.58		0.19			0.87		0.33		0.32			0.87		0.81			0.19
mean SBP [ms]	0.16		0.93			0.75		0.06		0.24			0.75		0.64			0.66
**Parasympathetic modulation**																		
RRI-RMSSD [ms]	0.22		0.33			0.87		0.90		0.13			0.17		0.52			0.56
RRI-HF powers [ms^2^]	0.40		0.50			0.63		0.90		0.30			0.51		0.66			0.72
**Sympathetic modulation**																		
RRI-LF powers [ms^2^]	0.11		0.24			0.52		0.32		0.39			0.87		0.45			0.72
SBP-LF powers [mmHg^2^]	0.19		0.87			0.34		0.02		0.57			0.05		1			0.81
**Total autonomic modulation**																		
RRI total powers [ms^2^]	0.14		0.29			0.52		0.42		0.46			1		0.45			0.81
RRI-SD [ms]	0.17		0.22			0.75		0.49		0.41			0.74		0.59			0.81
RRI-CV [%]	0.20		0.25			0.52		0.27		0.46			0.74		0.59			0.41
**Sympatho-vagal balance**																		
RRI-LF/HF-ratio	0.22		0.15			0.75		0.08		1			0.22		0.59			0.41

The table was split for better readability with autonomic parameters shown in the upper half and *p*-Values shown in the lower half. *<10 a*, disease duration under 10 years; *>10 a*, disease duration more than 10 years; *rTLE*, right TLE; *lTLE*, left TLE; *Ct*, controls; *RRI*, RR interval; *SBP*, systolic blood pressure; *RMSSD*, root mean square of successive differences; *HF*, high frequency; *LF*, low frequency; *SD*, standard deviation; *CV*, coefficient of variation. *<1/M*, less than 1 seizure per month; *>1/M*, more than 1 seizure per month.

**Table 4:** Biosignals and parameters of autonomic cardiovascular modulation according to antiseizure medication.

	Patients with no ASM (n = 1)	Patients with 1 ASM (n = 6)	Patients with 2 ASM (n = 16)	Patients with 3 ASM (n = 8)
**Biosignals**, Median (IQR)				
mean RRI [ms]	725.2	734.4 (580.7–832.1)	771.3 (714.6–831.7)	843.9 (649.0–926.9)
mean SBP [ms]	112.5	114.2 (103.9–120.3)	126.7 (109.3–139.0)	127.1 (118.0–132.8)
**Parasympathetic modulation**				
RRI-RMSSD [ms]	7.7	16.7 (9.0–24.8)	23.1 (8.6–33.5)	17.1 (9.3–23.4)
RRI-HF powers [ms^2^]	18.2	152.5 (34.8–470.0)	187.0 (44.0–469.1)	19.2 (11.7–41.2)
**Sympathetic modulation**				
RRI-LF powers [ms^2^]	88.2	633.3 (250.1–967.6)	575.2 (228.3–1913.7)	466.8 (157.8–764.0)
SBP-LF powers [mmHg^2^]	3.3	13.2 (6.1–35.3)	18.0 (7.6–23.4)	11.2 (6.2–21.0)
**Total autonomic modulation**				
RRI total powers [ms^2^]	106.4	794. 6 (284.8–1616.7)	824.5 (301.6–2.387.3)	828.0 (191.1–1009.3)
RRI-SD [ms]	12.1	30.4 (18.2–39.8)	32.6 (19.0–56.6)	30.8 (14.4–34.0)
RRI-CV [%]	1.7	4.5 (2.1–5.6)	4.2 (2.8–6.9)	3.3 (2.3–4.7)
**Sympatho-vagal balance**				
RRI-LF/HF-ratio	1.5	2.9 (1.7–4.6)	3.9 (1.6–8.3)	2.9 (2.0–5.4)

*ASM*, antiseizure medication; *RRI*, RR interval; *SBP*, systolic blood pressure; *RMSSD*, root mean square of successive differences; *HF*, high frequency; *LF*, low frequency; *SD*, standard deviation; *CV*, coefficient of variation.

**Table 5:** Biosignals and parameters of autonomic cardiovascular modulation according to age.

		**Controls < 30 a** (n = 10)		**Controls > 30 a** (n = 20)		**Pat. < 30 a with rTLE** (n = 3)		**Pat. > 30 a with rTLE** (n = 9)		**Pat. < 30 a with lTLE** (n = 8)		**Pat. > 30 a with lTLE** (n = 11)	
**Biosignals**, Median (IQR)													
mean RRI [ms]		767.3 (683.1–841.6)		754.1 (728.7–927.0)		709.2 (-)		730.6 (647.9–881.2)		790.4 (600.1–837.6)		781.3 (743.8–893.3)	
mean SBP [ms]		124.4 (120.8–137.6)		129.3 (122.2–140.0)		114.4 (-)		125.4 (114.2–137.4)		125.6 (117.3–131.4)		120.2 (107.9–128.1)	
**Parasympathetic modulation**													
RRI-RMSSD [ms]		25.3 (20.4–45.5)		21.2 (14.7–29.5)		35.3 (-)		15.6 (7.4–23.1)		21.8 (18.7–25.5)		15.5 (7.7–32.1)	
RRI-HF powers [ms^2^]		323.7 (202.6–834.6)		155.3 (40.1–277.3)		500.2 (-)		63.1 (20.8–270.3)		314.8 (107.1–398.7)		91.2 (34.5–464.8)	
**Sympathetic modulation**													
RRI-LF powers [ms^2^]		1620.8 (722.1–3979.9)		21.2 (14.7–29.5)		1657.8 (-)		244.4 (85.7–702.6)		663.3 (443.3–1285.9)		424.9 (180.5–1999.0)	
SBP-LF powers [mmHg^2^]		15.7 (12.5–46.2)		155.3 (40.1–277.3)		26.2 (-)		10.7 (5.8–23.0)		17.5 (8.1–25.0)		11.9 (6.6–20.2)	
**Total autonomic modulation**													
RRI total powers [ms^2^]		1835.6 (1039.7–5250.6)		21.2 (14.7–29.5)		2158.0 (-)		307.5 (125.0–1072.2)		996.5 (827.0–1393.1)		588.4 (219.0–2463.7)	
RRI-SD [ms]		43.8 (34.0–77.1)		155.3 (40.1–277.3)		57.4 (-)		19.2 (12.5–34.3)		34.1 (30.4–40.9)		26.3 (16.7–54.2)	
RRI-CV [%]		6.1 (4.9–8.3)		3.7 (3.1–5.3)		7.1 (-)		2.4 (2.0–4.0)		4.3 (3.4–5.1)		3.5 (2.4–6.5)	
**Sympatho-vagal balance**													
RRI-LF/HF-ratio		4.7 (2.8–7.9)		6.3 (1.8–10.5)		2.3 (1.7–3.7)		2.3 (1.7–3.7)		4.7 (1.6–5.7)		2.7 (1.3–13.8)	
	***p*****-Values** (Ct < 30 a vs. >30 a)		***p*****-Values** (Ct vs. rTLE < 30 a)		***p*****-Values** (Ct vs. rTLE > 30 a)		***p*****-Values** (Ct vs. lTLE < 30 a)		***p*****-Values** (Ct vs. lTLE > 30 a)		***p*****-Values** (r vs. lTLE < 30 a)		***p*****-Values** (r vs. lTLE > 30 a)
**Biosignals**, Median (IQR)													
mean RRI [ms]	0.57		0.50		0.32		0.79		0.71		0.68		0.43
mean SBP [ms]	0.60		0.61		0.40		0.53		0.05		0.68		0.31
**Parasympathetic modulation**													
RRI-RMSSD [ms]	0.14		0.61		0.13		0.29		0.51		0.31		0.47
RRI-HF powers [ms^2^]	0.02		0.74		0.51		0.29		0.59		0.31		0.85
**Sympathetic modulation**													
RRI-LF powers [ms^2^]	0.01		0.61		0.07		0.03		0.64		0.22		0.34
SBP-LF powers [mmHg^2^]	0.57		0.61		0.17		0.79		0.23		0.41		0.91
**Total autonomic modulation**													
RRI total powers [ms^2^]	0.01		0.61		0.09		0.04		0.62		0.41		0.47
RRI-SD [ms]	0.01		0.87		0.08		0.08		0.65		0.31		0.47
RRI-CV [%]	<0.01		0.87		0.08		0.03		0.48		0.10		0.31
**Sympatho-vagal balance**													
RRI-LF/HF-ratio	0.76		0.61		0.11		0.42		0.54		0.41		0.62

The table was split for better readability with autonomic parameters shown in the upper half and *p*-Values shown in the lower half.  *<30 a*, under 30 years of age; *>30 a*, more than 30 years of age; *rTLE*, right TLE; *lTLE*, left TLE; *Ct*, controls; *RRI*, RR interval; *SBP*, systolic blood pressure; *RMSSD*, root mean square of successive differences; *HF*, high frequency; *LF*, low frequency; *SD*, standard deviation; *CV*, coefficient of variation.

## Data Availability

Data are available on request from the authors via mail to the corresponding authors, subject to approval and a data sharing agreement.

## Abbreviations

The following abbreviations are used in this manuscript:

ASM	antiseizure medication
TLE	temporal lobe epilepsy
RRI	R-R-interval
BP	blood pressure
CV	coefficient of variation
RMSSD	root mean square of successive differences
LF	low frequency
HF	high frequency
SBP	systolic blood pressure
SD	standard deviation
SeLEAS	self-limited epilepsy with autonomic seizures
SUDEP	sudden unexpected death in epilepsy
EEG	electroencephalography
rTLE	right temporal lobe epilepsy
lTLE	left temporal lobe epilepsy
DBP	diastolic blood pressure
Ct	controls
HRV	heart rate variability
